# Unveiling Differential Responses of Granulocytes to Distinct Immunostimulants with Implications in Autoimmune Uveitis

**DOI:** 10.3390/biomedicines12010019

**Published:** 2023-12-20

**Authors:** Roxane L. Degroote, Adrian Schmalen, Stefanie M. Hauck, Cornelia A. Deeg

**Affiliations:** 1 Chair of Physiology, Department of Veterinary Sciences, LMU Munich, D-82152 Martinsried, Germany; r.degroote@lmu.de (R.L.D.); adrian.schmalen@tiph.vetmed.uni-muenchen.de (A.S.); 2 Metabolomics and Proteomics Core, Helmholtz Center Munich, German Research Center for Environmental Health, D-80939 Munich, Germany; stefanie.hauck@helmholtz-munich.de

**Keywords:** ERU, PMN, granulocyte, innate immune cell activation, granulocyte heterogeneity, differential proteomics, pathway analysis, IL8, PMA, LPS

## Abstract

The perception of circulating granulocytes as cells with a predetermined immune response mainly triggered by pathogens is evolving, recognizing their functional heterogeneity and adaptability, particularly within the neutrophil subset. The involvement of these cells in the pathophysiology of autoimmune uveitis has become increasingly clear, yet their exact role remains elusive. We used an equine model for autoimmune-mediated recurrent pan-uveitis to investigate early responses of granulocytes in different inflammatory environments. For this purpose, we performed differential proteomics on granulocytes from healthy and diseased horses stimulated with IL8, LPS, or PMA. Compared to healthy horses, granulocytes from the recurrent uveitis model significantly changed the cellular abundance of 384 proteins, with a considerable number of specific changes for each stimulant. To gain more insight into the functional impact of these stimulant-specific proteome changes in ERU pathogenesis, we used Ingenuity Pathway Analysis for pathway enrichment. This resulted in specific reaction patterns for each stimulant, with IL8 predominantly promoting Class I MHC-mediated antigen processing and presentation, LPS enhancing processes in phospholipid biosynthesis, and PMA, clearly inducing neutrophil degranulation. These findings shed light on the remarkably differentiated responses of neutrophils, offering valuable insights into their functional heterogeneity in a T-cell-driven disease. Raw data are available via ProteomeXchange with identifier PXD013648.

## 1. Introduction

With little to no capacity for regeneration, the ocular microenvironment needs protection against excessive inflammation and is considered immune privileged [1,2,3]. However, in autoimmune uveitis, detrimental immune reactions overcome said privilege, threatening the vision of patients (reviewed in [4]). Besides a dysregulated T cell response, the involvement of innate immune cells in the pathogenesis of autoimmune uveitis has become evident [5,6,7]. Their exact role in the pathology of autoimmune uveitis, however, remains elusive. The effector cells driving autoimmune uveitis from the periphery are easily accessible through blood withdrawal, contrary to eye tissue, which can only be obtained through invasive procedures risking vision loss. Therefore, these cells are useful for investigations on the molecular mechanisms contributing to disease pathogenesis and may serve as valuable targets for causal diagnostic therapeutic approaches.

Equine recurrent uveitis (ERU) is a leading cause of blindness among horses worldwide [8,9]. It is characterized by recurring episodes of painful inflammation within inner eye structures, which increase in severity over time [8,9,10]. Onset, progression, and pathogenesis of ERU are similar to autoimmune uveitis in humans [11,12,13,14,15,16]. Further, the immune system per se shows a strong resemblance between said species [17]. These similarities together with the availability of equine ocular tissue and peripheral blood cells from animals without systemic treatment for disease renders the horse a valuable model organism to study autoimmune-mediated recurrent pan-uveitis.

Using this model, a pre-activated state of circulating granulocytes was previously shown in ERU-afflicted horses, indicating an active role of these innate immune cells instead of merely acting as passive bystanders in disease pathogenesis [18]. Similarly, a more readily activated feature of equine innate immune cells was also observed in other T-cell-driven disorders [19]. Further, we detected that granulocytes show a very specific response to different stimuli in vitro, which points to functional heterogeneity and finely tuned activation of downstream innate immune response depending on the inflammatory environment [20,21].

Since early influx of granulocytes into ocular tissue has been shown in experimental autoimmune uveitis in mice and rats [5,6,7,22,23,24], and also in horses with ERU [25], insights into initial granulocyte activation mechanisms may help to specifically target and restrict or at least interfere with granulocyte activity in early stages of disease. For this, it is important to know the diverse reaction patterns of granulocytes in different inflammatory environments. These can be simulated in vitro by several stimulatory agents. For instance, interleukin-8 (IL8) is a cytokine that acts as a potent chemoattractant that specifically promotes recruitment and activation of human granulocytes through chemokine receptors CXCR1 and CXCR2, thereby contributing to an inflammatory response [26]. As a component of the outer membrane of gram-negative bacteria, lipopolysaccharide (LPS) activates granulocytes by binding to toll-like receptor 4, thereby increasing cell surface adhesion molecules and vascular crawling [27]. Phorbol 12-myristate 13-acetate (PMA), on the other hand, triggers exocytosis, release of reactive oxygen species, and formation of neutrophil extracellular traps through direct activation of protein kinase C [28]. Since these stimuli trigger granulocyte activation in different ways, which simulate different inflammatory environments, we analyzed the early response of granulocytes from ERU-afflicted horses to IL8, LPS, and PMA, with a prospect of potentially new approaches in diagnostics and therapy of autoimmune-mediated recurrent pan-uveitis on a molecular level.

## 2. Materials and Methods

### 2.1. Sample Processing

Granulocytes were freshly obtained from heparinized (50 I.U./mL blood, Ratiopharm, Ulm, Germany) venous whole blood of three healthy horses and three horses with ERU. The healthy horses belong to the Equine Clinic at Ludwig-Maximilians-University Munich. Horses with ERU were patients awaiting therapeutic procedure. ERU was diagnosed by experienced clinicians from the Equine Clinic at Ludwig-Maximilians-University Munich and was based on typical clinical signs of uveitis, along with a documented history of multiple episodes of inflammation of the affected eye [29]. Blood withdrawal from ERU horses was performed in the course of diagnostics and therapy, in quiescent stage of disease. Blood withdrawal from healthy horses was performed by experienced clinicians from the Equine Clinic at Ludwig-Maximilians-University Munich and was permitted by the local authority (Regierung von Oberbayern, Permit number: ROB-55.2-2532.Vet_03-22-37).

After rough sedimentation of erythrocytes, granulocytes were isolated from blood plasma by density gradient centrifugation (room temperature, 350× *g*, 25 min, brake off) with Ficoll-Paque PLUS separating solution (density 1.077 g/mL; Cytiva Life Sciences, Freiburg, Germany). This procedure yielded separation into four layers: a top layer of blood plasma, a second layer with mostly peripheral blood mononuclear cells, a third layer of Ficoll-Paque PLUS separating solution, and a bottom layer containing mainly red blood cells and granulocytes [30]. After removal of the three top layers, cells from the bottom layer were carefully washed (4 °C, 400× *g*, 10 min) in cold PBS (DPBS devoid of CaCl_2_ and MgCl_2_; Gibco/ThermoFisher Scientific, Schwerte, Germany), and residual erythrocytes were removed by 30 s sodium chloride (0.2% NaCl) lysis. The isotonicity of samples was restored through the addition of equal parts 1.6% NaCl. The remaining granulocytes were then washed (4 °C, 400× *g*, 10 min) and resuspended in PBS with 0.2% glucose.

From each granulocyte specimen, we prepared aliquot portions of 6 × 10^5^ cells/500 µL, which were separately stimulated with recombinant equine interleukin-8 (IL8; Kingfisher Biotech; 1 ng/mL), phorbol 12-myristate 13-acetate (PMA; Sigma-Aldrich/Merck, Darmstadt, Germany; 5 µg/mL), and lipopolysaccharide (LPS; Sigma-Aldrich/Merck, Darmstadt, Germany; 5 µg/mL) for 30 min in a CO_2_ incubator (37 °C, 5% CO_2_). After stimulation, the volume of each aliquot was adjusted to 1 mL by adding PBS with 0.2% glucose. Cells were then pelleted (4 °C, 2300× *g*, 10 min) and stored at −20 °C. Prior to mass spectrometric analysis, granulocytes were thawed and lysed in urea buffer (8 M urea in 0.1 M Tris/HCl pH 8.5), and protein concentration was determined with Bradford protein assay [31].

### 2.2. Mass Spectrometric Analysis

From each sample, 10 µg of total protein was digested with LysC and trypsin by filter-aided sample preparation (FASP), as previously described [32]. Acidified eluted peptides were analyzed in a data-dependent mode on a QExactive HF mass spectrometer (Thermo Fisher Scientific, Dreieich, Germany) online coupled to a UItimate 3000 RSLC nano-HPLC (Dionex/ Thermo Fisher Scientific, Dreieich, Germany). Samples were automatically injected and loaded onto the C18 trap cartridge and after 5 min, they were eluted and separated on the C18 analytical column (nanoEase MZ HSS T3, 100 Å, 1,8 µm, 75 µm × 250 mm; Waters, Eschborn, Germany) by a 95-min non-linear acetonitrile gradient at a flow rate of 250 nL/min. MS spectra were recorded at a resolution of 60,000 with an automatic gain control (AGC) target of 3e^6^ and a maximum injection time of 30 ms from 300 to 1500 m/z. From the MS scan, the 10 most abundant peptide ions were selected for fragmentation via HCD with a normalized collision energy of 27, an isolation window of 1.6 m/z, and a dynamic exclusion of 30 s. MS/MS spectra were recorded at a resolution of 15,000 with an AGC target of 1e^5^ and a maximum injection time of 50 ms. Unassigned charges and charges of +1 and > +8 were excluded from precursor selection.

### 2.3. Data Processing and Label-Free Quantification

Proteome Discoverer 2.5 software (version 2.5.0.400; Thermo Fisher Scientific, Dreieich, Germany) was used for peptide and protein identification via a database search (Sequest HT search engine) against the Ensembl Horse protein database (version 3.0, http://ensembl.org (accessed on 3 November 2023)). The database search was performed with full tryptic peptide specificity, allowing for up to one missed tryptic cleavage site. The precursor mass tolerance was 10 ppm, and the fragment mass tolerance was 0.02 Da. Carbamidomethylation of cysteine was set as static modification. Dynamic modifications included deamidation of asparagine and glutamine, oxidation of methionine (M), and a combination of M loss with acetylation on the protein N-terminus. Peptide spectrum matches and peptides were validated with the Percolator algorithm [33]. Only the top-scoring hits for each spectrum were accepted with a false discovery rate (FDR) < 1% (high confidence). The final list of proteins satisfying the strict parsimony principle included only protein groups passing an additional protein confidence filter FDR < 5% filter (target/decoy concatenated search validation).

Quantification of proteins was based on intensity values (at RT apex) for the top three unique peptides per protein. Peptide abundance values were normalized on the total peptide amount. Protein abundances were calculated as the average of the three most abundant (Top 3 N) unique peptides. Missing values were replaced by low abundance imputation from the lowest 5% of detected abundance values. These protein abundances were used for the calculation of enrichment ratios of proteins per treatment comparison. Significance of the ratios was tested using a background-based *t*-test with correction for multiple testing according to Benjamini-Hochberg (adjusted *p*-value) [34,35].

### 2.4. Data Analysis

Data were analyzed with the use of Ingenuity Pathway Analysis (IPA; Qiagen, Hilden, Germany, https://digitalinsights.qiagen.com/ (accessed on 7 November 2023)) [36]. Ensembl stable protein identifier and Entrez Gene identifier were used to map equine proteins to protein identifiers compatible with the Ingenuity Knowledge Base, resulting in 2673 mapped and 638 unmapped proteins. Analysis was based on the abundance ratios, the abundance ratio *p*-values, and the abundance ratio adjusted *p*-values. IPA Core Analysis was performed with a significance threshold of –log10(*p*-value) > 1.3 (=*p*-value < 0.05) and an expression fold change threshold of −2 for downregulated genes and 2 for upregulated genes. Relaxed tissue filtering was set to granulocytes. Z-score describes prediction of activation (+ values) or inhibition (−values) of enriched pathway. From data input, IPA evaluates overrepresentation of proteins in canonical pathways, molecular patterns of diseases, or other cellular functions, which are deposited in the Ingenuity Knowledge Base, as previously described [36]. This allows insight into possible physiological effects of upstream molecules on these proteins and allocation to downstream pathways.

Volcano plots were created in R (version 4.3.1; R Core Team (2023), Vienna, Austria, https://www.R-project.org/ (accessed on 8 November 2023)) [37] with the packages ggplot2 (version 3.4.2) [38] and ggrepel (version 0.9.3) [39]. Gene names for the data point labels of the volcano plot were obtained from the protein description of the Ensembl Horse database. Where possible, gene names of uncharacterized proteins were translated into human gene names using the Ensembl Biomart database together with the equine Ensembl stable protein identifiers (https://www.ensembl.org/info/data/biomart; exported on 8 August 2023). Venn diagram was generated with the open-source tool: http://bioinformatics.psb.ugent.be/webtools/Venn/ (accessed on 8 November 2023).

## 3. Results

### 3.1. In an Inflammatory Uveitis Model, Granulocytes Show Distinct Protein Changes Dependent on Treatment with IL8, LPS, or PMA

**Figure 1:**
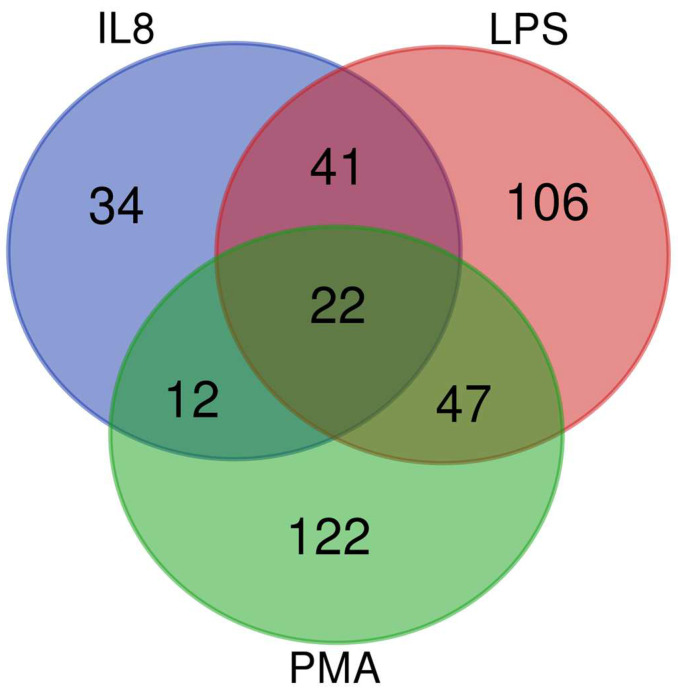
Distinct protein expression in granulocytes of ERU-afflicted horses following treatment with various cytokines: Venn diagram of differentially expressed proteins (adjusted *p*-value < 0.05) in granulocytes derived from ERU horses compared to healthy controls, treated as indicated. After IL8 stimulation, a total of 109 proteins showed changed abundance (blue circle). Of these, 34 were uniquely changed in IL8-stimulated cells, whereas 75 were also changed in the other samples. Stimulation with LPS yielded abundance changes of 216 proteins (red circle), of which 106 were solely changed for this stimulant and 110 were shared. PMA stimulation resulted in 203 differentially abundant proteins (green circle), of which 122 were not changed in the samples incubated with the other two stimulants and 81 were changed in all three stimulants.

Granulocytes may play a more crucial role in the pathogenesis of ERU than initially thought [18,20,25,40,41,42,43]. While pre-activation of granulocytes in ERU has been previously implied [18], it remains unclear how this affects their behavior in the stimulatory environment of ERU eyes. Thus, we compared the effects of three different stimuli on granulocytes from healthy horses and horses with ERU. This allowed us to elucidate the inherent pre-activated status of ERU-derived granulocytes and to detect consequent changes in their reaction in different inflammatory environments on a molecular level. We quantitatively analyzed the proteome of granulocytes derived from healthy and ERU-afflicted horses following stimulation with IL8, LPS, or PMA, respectively for 30 min. Using this method, we quantified 2861 proteins in the lysates of equine granulocytes (Supplementary Table S1). Of these, 384 proteins showed significant (adjusted *p*-value < 0.05) abundance differences in ERU (Figure 1). Specifically, IL8 stimulation yielded 109 differentially abundant proteins, of which 60 were less abundant and 49 were more abundant in ERU samples. LPS induced abundance differences of 216 proteins, which included 104 proteins with lower expression levels and 112 with higher abundance. Stimulation with PMA rendered a total of 203 proteins with changed abundance, among which 70 proteins were lower and 133 were more abundant in ERU (Supplementary Table S1, Figure 1).

The analysis of differentially expressed granulocyte-derived proteins per treatment group revealed protein abundance changes for 34 proteins only from IL8 stimulation (Figure 1, blue circle), 106 differentially expressed proteins specifically following treatment with LPS (Figure 1, red circle), and 122 proteins that only showed differential abundance after stimulation with PMA (Figure 1, green circle). Twenty-two differentially expressed proteins were common to all three treatment groups, indicating proteins that were consistently altered in response to stimulation in granulocytes derived from ERU-afflicted horses, regardless of the specific stimulus (Figure 1, overlap area of all three circles). Of all the differentially abundant proteins in our dataset, 8.9% were shared between IL8- and PMA-stimulated samples, 16.4% were shared between samples stimulated with IL8 and LPS, and 18% overlap was detected after treatment with LPS and PMA. This shows a predominantly specific change in protein abundance for each stimulant rather than mostly similar reaction patterns. In particular, with regard to LPS and PMA, which are both known to induce similar processes in granulocytes of other species [27,28], the small amount of shared differentially abundant proteins after stimulation of equine granulocytes underlines functional heterogeneity and finely tuned response pattern of these innate immune cells in diseased state.

### 3.2. Granulocytes Show Distinct Functional Heterogeneity in Different Inflammatory Environments

**Figure 2:**
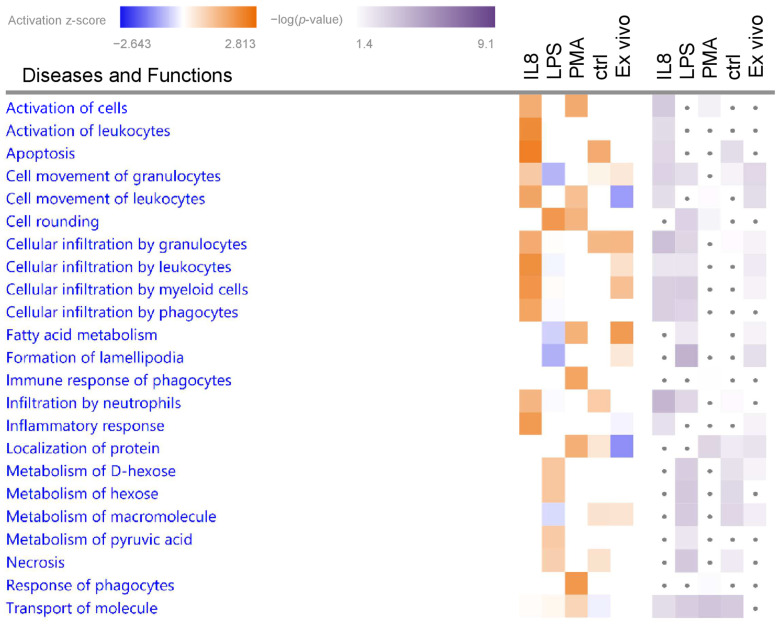
Top biological functions enriched in ERU-afflicted granulocytes following short-term treatment with various stressors. Granulocytes from ERU horses and healthy controls were treated as indicated for 30 min. Biological functions enriched in ERU-derived granulocytes compared to the corresponding healthy controls were identified by IPA. Heatmaps of top biological functions with z-score > 2 and *p*-value < 0.05 for at least one of the stimulants are shown in alphabetical order. Orange squares indicate a positive activation z-score, whereas blue squares indicate a negative z-score. White squares indicate no activation prediction for the corresponding pathway. Shades of purple indicate the –(log10) *p*-value with darker squares being more significant. Dots indicate *p*-values > 0.05.

**Figure 3:**
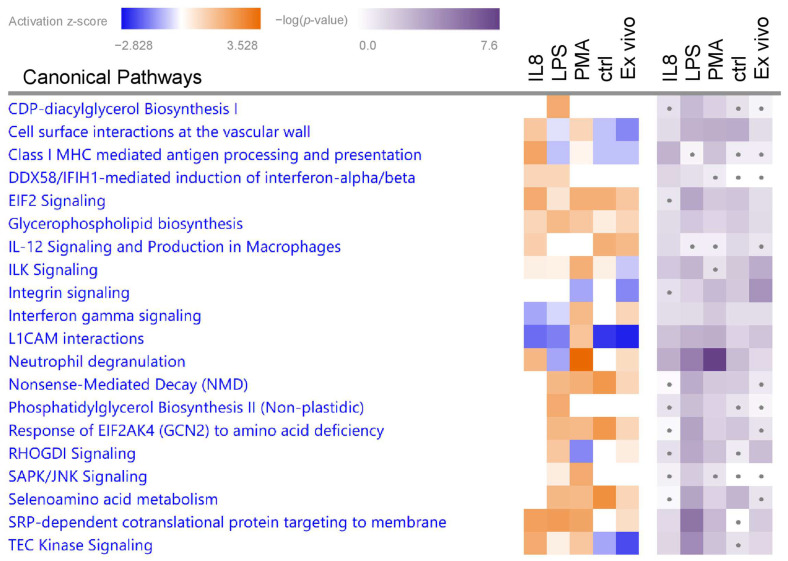
Top canonical pathways enriched in ERU-afflicted granulocytes following short-term treatment with various stressors. Granulocytes from ERU horses and healthy controls were treated as indicated for 30 min. Canonical pathways enriched in ERU-derived granulocytes compared to the corresponding healthy controls were identified by IPA. Heatmaps of top canonical pathways with z-score > 2 and *p*-value < 0.05 for at least one of the stimulants are shown in alphabetical order. Orange squares indicate a positive activation z-score, whereas blue squares indicate a negative z-score. For white squares, no z-score could be calculated. Shades of purple indicate the –(log10) *p*-value, with darker squares being more significant. Dots indicate *p*-values > 0.05.

To gain better insight into the functional impact of the differentially abundant proteins from granulocytes in ERU pathogenesis and to compare possible differences between stimuli, we used IPA for pathway and biological function enrichment analysis (Supplementary Table S2). We focused on significantly (*p* < 0.05) enriched canonical pathways and biological functions with predicted activation (positive z-score > 1.5 for biological functions or >2 for canonical pathways) (Figure 2 and Figure 3, Table 1 and Table 2).

#### 3.2.1. Biological Functions in Granulocytes with Predicted Activation

Biological function enrichment analysis of all stimulants (Supplementary Table S2) revealed “Activation of cells” as significantly enriched in both the IL8- and PMA-stimulated samples, but not in the samples stimulated with LPS (Figure 2, Table 1). Analysis of granulocyte proteome changes after IL8 stimulation resulted in a total of nine significantly enriched biological functions, of which “Apoptosis” showed the highest z-score for activation. Further, IPA allocated differentially abundant proteins from the IL8 dataset to the functions “Activation of leukocytes” and “Inflammatory response”, as well as the category “cellular movement and immune cell trafficking”, in which five of the enriched biological functions were clustered. The biological function “Cell rounding” was allocated to differentially abundant proteins from LPS-stimulated granulocytes, whereas PMA-induced protein abundance changes were associated to “Response of phagocytes” and “Immune response of phagocytes” (Figure 2, Table 1).

#### 3.2.2. Canonical Pathways in Granulocytes with Predicted Activation

**Figure 4:**
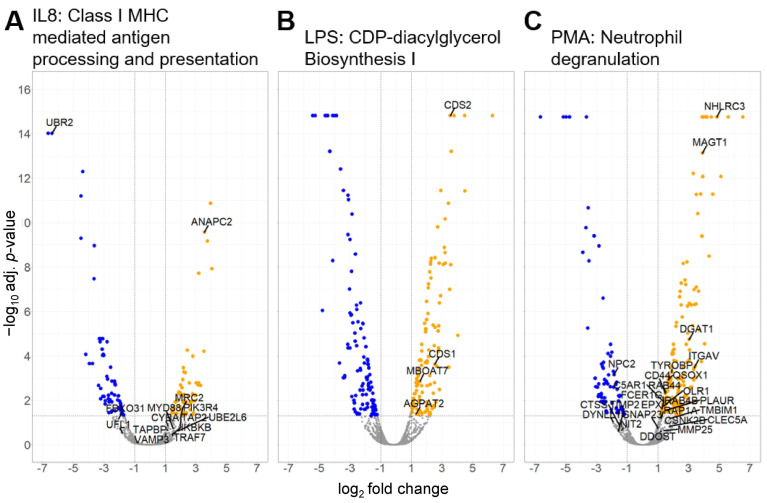
Volcano plot of all identified proteins from ERU-afflicted granulocytes following short-term incubation with different stressors. Granulocytes derived from ERU horses and healthy controls were treated as indicated for 30 min. Proteins with significant changes in their abundance ratio (±log_2_(1) fold expression, adjusted *p*-value ≤ 0.05) are colored, with upregulated proteins depicted as orange dots, while down-regulated proteins are colored blue. Grey dots show proteins with no significant abundance changes. One significantly enriched pathway per treatment identified by IPA was chosen, and the associated proteins were labeled with their gene symbol. Class I MHC-mediated antigen processing and presentation pathway was enriched in IL8-treated ERU granulocytes (**A**). CDP-diacylglycerol Biosynthesis I pathway was enriched in LPS-treated ERU granulocytes (**B**). Neutrophil degranulation pathway was enriched in PMA-treated ERU granulocytes (**C**).

Stimulation of granulocytes with IL8 yielded 99 significantly enriched canonical pathways (Supplementary Table S2), of which three pathways were predicted to be activated with a z-score larger than 2 (Figure 3, Table 2). From these, we selected the pathway with the most significant *p*-value and the highest z-score for further analysis. Although the pathway “SRP-dependent cotranslational protein targeting to membrane” was enriched with a higher z-score, we chose the pathway “Class I MHC-mediated antigen processing and presentation” for further analysis of IL8-stimulated cells due to a better *p*-value and because antigen cross-presentation is a highly interesting process in ERU since it might enhance inflammation of a diseased retina (Figure 3 and Figure 4A, Table 2). Differentially abundant granulocyte proteins resulting from treatment with LPS were allocated to 145 significantly enriched pathways (Supplementary Table S2), including three pathways with a z-score larger than 2 (Figure 3, Table 2). Amongst these were two pathways associated with phospholipid biosynthesis: “CDP-diacylglycerol Biosynthesis I” and “Phosphatidylglycerol Biosynthesis II (Non-plastidic)” (Figure 3, Table 2). Phosphatidylinositols are involved in the regulation of granulocyte apoptosis [44] and neutrophil directional movement [45]. Since the synthesis of phosphatidylinositols is part of this pathway, we chose to further investigate the activation of the “CDP-diacylglycerol Biosynthesis I” pathway in ERU granulocytes (Figure 3 and Figure 4B and Table 2). Finally, proteome changes after PMA stimulation of granulocytes clustered to 108 significantly enriched pathways (Supplementary Table S2). Two pathways were predicted to be activated with a z-score larger than two (Figure 3, Table 2). One of these two pathways was the “neutrophil degranulation pathway”, which we chose for further analysis (Figure 3 and Figure 4C, Table 2). The majority of the molecules involved in the selected pathways allocated to the three different stimulants were upregulated (Figure 4, orange dots), confirming the significant activation as calculated by IPA. Furthermore, no overlap in proteins associated with the three top enriched canonical pathways was detected (Table 2), indicating the specific effect of the different stimulants on granulocytes of ERU horses.

**Figure 5:**
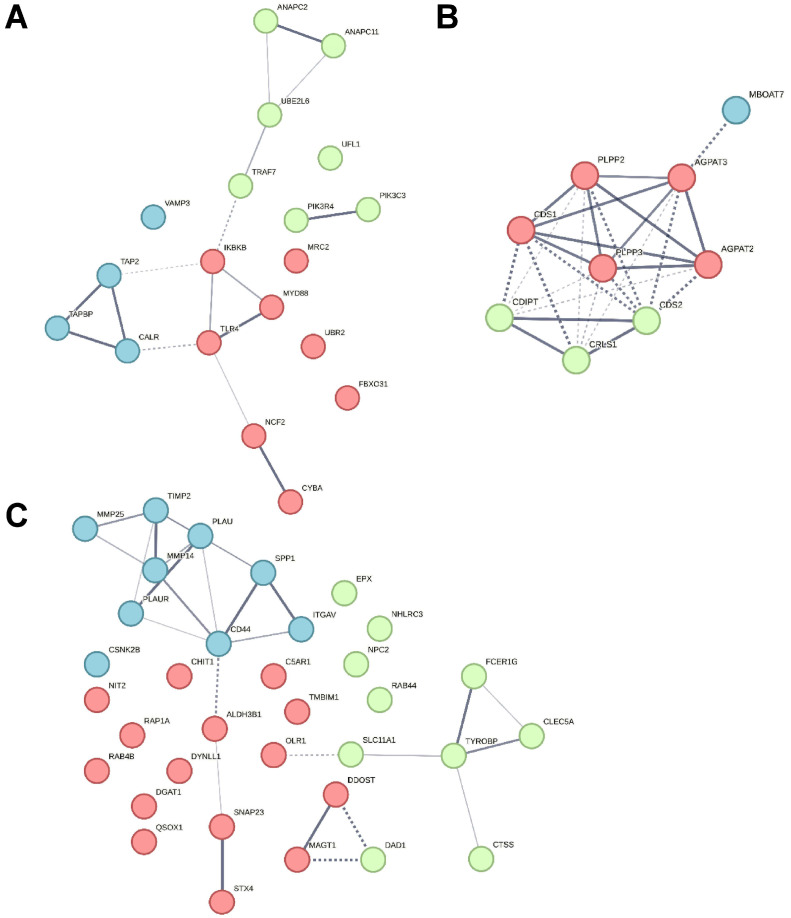
Protein interaction network of enriched canonical pathways in granulocytes from ERU horses following treatment with various stressors. Granulocytes derived from ERU horses and healthy controls were treated with IL8, LPS, or PMA, respectively for 30 min. Subsequently, the proteome was analyzed, and pathway enrichment analysis was performed using IPA. Per treatment, the most significantly (*p*-value < 0.05) enriched pathway with a z-score threshold > 2 was chosen. Protein interaction networks were generated using the String database for proteins associated with the “Class I MHC-mediated antigen processing and presentation” pathway for IL8-treated granulocytes (**A**), “CDP-diacylglycerol Biosynthesis I” pathway for LPS-treated granulocytes (**B**), and “Neutrophil degranulation” pathway for PMA-treated granulocytes (**C**). Kmeans clustering was performed to sort each network into three distinct clusters, which were colored blue, green or red.. Edges between the proteins show interaction, with line thickness indicating confidence of interaction. Dashed lines show interactions between proteins from different clusters.

To gain deeper insights into protein-protein interactions of the differentially abundant proteins allocated to each selected canonical pathway, we subsequently constructed an interaction network using the String Database (version 12.0; available free of charge at: https://string-db.org/ (accessed on 9 November 2023)) with these proteins (Figure 5). One additional level of predicted functional partners was allowed during network generation, and kmeans clustering was performed to sort each interaction network into three distinct clusters.

Proteins Antigen peptide transporter 2 (*TAP2*), Tapasin (*TAPBP*), and Calreticulin (*CALR*) in the blue cluster from the Class I MHC-mediated antigen processing and presentation pathway are associated with the MHC class I peptide loading complex [46] (Figure 5A). The green cluster contained proteins linked to protein degradation via the ubiquitin-proteasome system, which generates peptides suitable for MHC class I antigen presentation. Protein degradation is predominantly promoted by ubiquitin-conjugating enzymes such as Ubiquitin/ISG15-conjugating enzyme E2 L6 (*UBE2L6*) and by ubiquitin ligases like Anaphase-promoting complex subunit 2 (*ANAPC2*) and subunit 11 (*ANAPC11*), which constitute the catalytic component of the anaphase-promoting complex [47] (Figure 5A). Immune regulatory proteins like Toll-like receptor 4 (*TLR4*), Myeloid differentiation primary response protein MyD88 (*MYD88*), and IkappaB kinase (*IKBKB*) [48] were part of the red cluster (Figure 5A).

The network of proteins allocated to the CDP-diacylglycerol Biosynthesis I pathway was highly interconnected with strong edges, indicating high confidence (Figure 5B). The proteins in this network are involved in the synthesis of glycerol lipids and metabolism of phosphatidylinositol (reviewed in [49]) (Figure 5B).

In the neutrophil degranulation pathway (Figure 5C), CD44 was the most interconnected node with six edges. CD44, a receptor for hyaluronic acid [50], was allocated to the blue cluster that also contained proteins involved in the remodeling of the extracellular matrix, such as Matrix Metalloproteinase-25 (*MMP25*) and its inhibitor Metalloproteinase inhibitor 2 (*TIMP2*) [51]. The green cluster contained receptor proteins like C-type lectin domain family 5, member A (*CLEC5A*), TYRO protein tyrosine kinase binding protein (*TYROBP*), and High affinity immunoglobulin epsilon receptor subunit gamma (*FCER1G*) but also the protease Cathepsin S (*CTSS*). These proteins are associated with leukocyte migration and persistent inflammation [52,53]. Proteins in the red cluster of the neutrophil degranulation network, such as Synaptosomal-associated protein 23 (*SNAP23*) which is involved in exocytosis [54], showed little interaction with a low number of edges (Figure 5C).

## 4. Discussion

The stigma of circulating granulocytes as terminally differentiated cells with a fixed immune response to stimuli mainly induced by pathogens is shifting to the acknowledgement of functional heterogeneity and plasticity, especially in the neutrophil population (reviewed in [55]). In this context, their active involvement in diseases driven by cells of the adaptive immune system, such as autoimmune uveitis, is especially interesting. While granulocytes have been previously shown to participate in the pathogenesis of recurrent autoimmune-mediated uveitis as disease mediators, their mode of action remains elusive [5,6,24,25,56]. To gain deeper insights into the early mechanisms behind heterogenic granulocyte activation and to further support the assertion of a pre-activated state in ERU, we used an equine model to investigate the reaction pattern of granulocytes to different stimuli on a molecular level.

ERU is an important disease among horses since it causes severe pain in the afflicted animals and substantially contributes to blindness among the horse population. When affecting both eyes, ERU will result in the death of the horse through euthanasia, since blind horses pose a great risk to themselves and their surroundings. Although various factors linked to the onset of ERU are still under discussion, an exact cause is yet to be determined. As with any animal model used for studies on diseases in humans, the transferability of obtained insights needs careful assessment. But despite the given differences [57,58], substantial similarities in both immune system function and composition [17,42,59,60,61] render the horse a highly promising model for autoimmune-mediated recurrent uveitis. This is further substantiated by several studies, which already demonstrated that adaptive as well as innate immune cells from horses can be valuable tools for studying pathological disorders in humans [15,17,62,63]. Nonetheless, the exact value of the horse as a model for diseases in humans needs to be substantiated by further investigations.

In this study, we reanalyzed previous examination of granulocytes derived from ERU-afflicted horses treated with IL8 [20], but expanded our analysis by two additional stimulatory agents, namely LPS and PMA, to obtain information on the reaction patterns of granulocytes in different inflammatory environments produced by the different stimuli. Additionally, we applied more stringent settings for pathway-enrichment analysis, by including a filter for cell type-associated proteins, as well as stricter significance- and expression-value thresholds. IL8 acts on leukocytes as a chemotactic agent and has been found to induce a type of activation that allows granulocytes to cross the retina-blood barrier [20]. Therefore, the activation of the biological function “cellular infiltration by granulocytes” in IL8-stimulated granulocytes was plausible, given the chemotactic properties of IL8 as a stimulant. While pathways associated with cellular movement induced by treatment with IL8 have been reproduced with our more stringent filtering settings, other cellular functions and pathways have not been described previously [20].

Granulocytes derived from healthy horses have been shown to regulate specific pathways in response to stimulation by IL8 and PMA [21]. Stimulation of granulocytes with LPS, on the other hand, did not lead to significant enrichment of any pathways in said study [21]. In contrast, we were able to detect that granulocytes derived from ERU horses showed significant and diverse pathway enrichment for all three stimulants. For LPS in particular, this might originate from the fact that granulocytes from ERU horses are more sensitive to stimulation as they are in a pre-activated state, allowing rapid reaction to stimuli [18,20].

IL8, PMA, as well as LPS, induced the activation of the pathway “SRP-dependent cotranslational protein targeting to membrane” in granulocytes from ERU horses. This pathway is important for proper protein localization and function [64]. It ensures the correct delivery of newly synthesized proteins with signal sequences to cellular membranes, such as the plasma membrane, granule membranes, or other granulocyte organelles [65]. Therefore, it is not surprising that this pathway was shown to be activated in ERU granulocytes, regardless of the stimulant used.

The predicted activation of the pathway “Class I MHC-mediated antigen processing and presentation” in granulocytes stimulated with IL8 is consistent with previous findings, describing this pathway as activated in ERU [18]. Although said pathway emerged in both IL8-stimulated and untreated granulocytes, allocated proteins differed: After IL8 stimulation of ERU granulocytes, differentially abundant proteins were those part of the MHC I peptide loading complex whereas previous analysis of untreated ERU cells showed proteins involved in lysosome acidification and phagosome-lysosome fusion to be more abundant [18]. This indicates a specific shift of focus from one part of the said pathway, which is generally activated in ERU, to a different part, which seems to be specifically activated after IL8 treatment of cells. Interestingly, certain subtypes of the human MHC class I allotype HLA-B27 are strongly associated with the development of anterior autoimmune uveitis [66]. However, the exact mechanism is part of an ongoing scientific discussion (for a summary see [67]). Under certain conditions, granulocytes may express or upregulate MHC class I molecules for cross-presentation [68,69]. This process describes the presentation of fragments derived from incorporated exogenous proteins. Granulocytes are phagocytic cells [70]. Consequently, uptake of exogenous antigens by phagocytosis and simultaneous induction of MHC class I might promote cross-presentation and thus, enhance the risk for molecular mimicry (reviewed in [71]). Molecular mimicry describes autoimmunity as a result of antigen presentation of an exogenous antigen that is similar to an endogenous self-antigen [72]. One such epitope might be a peptide derived from the nitrogenase protein of *Klebsiella* that shares six consecutive amino acids with HLA-B27 [73]. Additionally, the expression of HLA-B27 might interfere with the composition of the microbiome, affecting immune responses and the development of autoimmune diseases [74,75,76]. Although the underlying mechanisms are poorly understood, it has been proposed that a divergent microbiome affects the permeability of the gut wall, promoting a systemic immune response [77]. While granulocytes derived from ERU-afflicted horses are pre-activated, this is most likely not due to a microbiome-dependent inflammation, since no significant alterations in the microbiome have been previously identified in the feces of ERU horses [78].

As mentioned beforehand, stimulation of granulocytes derived from ERU horses with LPS yielded the significant enrichment of several pathways and functions, amongst others “cell rounding”. Cell rounding in neutrophils may specifically refer to changes in their morphology during the process of diapedesis through barriers in order to reach the site of infection or tissue damage. Neutrophils undergo a series of shape changes during diapedesis, and rounding is one of the characteristic morphological alterations observed [79]. As indicated for stimulation with IL8, processes associated with directed cell movement seem to continuously be more readily activated in granulocytes from ERU horses compared to controls. Besides the significant activation of the biological process “cell rounding”, the two canonical pathways “CDP-diacylglycerol Biosynthesis I” and “Phosphatidylglycerol Biosynthesis II (Non-plastidic)” were also activated after LPS stimulation. Both pathways fall into the category of phospholipid biosynthesis and metabolism, a process that is influenced by inflammatory signals and cytokines released during an immune response, to meet the cell’s changing needs. When granulocytes are activated, there is an increased demand for membrane components, including phospholipids, to support processes like phagocytosis, exocytosis, and the formation of neutrophil extracellular traps [80]. In phospholipid-dependent signaling, phospholipid hydrolysis is mediated by many different phospholipases, and their synchronized activity plays a crucial role in initiating cell activation (reviewed in [81]). In mouse granulocytes, the phospholipase Cγ subtypes, which are activated by tyrosine kinase–linked receptors, play a pivotal role in supporting neutrophil respiratory burst, phagocytosis, adhesion, and cell migration [82]. Interestingly, we detected a very prominent association of LPS-induced protein changes to several pathways associated with phospholipid biosynthesis in granulocytes from ERU horses. In granulocytes from healthy horses, on the other hand, this connection was not made, despite these processes being required for activation [21]. The reason behind this may lay in the fact that we used a very short stimulation time and compared to granulocytes from healthy horses, ERU-derived cells show a more rapid reaction to stimulants due to their pre-activated phenotype.

Among the granulocyte subpopulations, neutrophils, in particular, release inflammatory mediators and proteolytic enzymes as part of their degranulation process, contributing to wanted antimicrobial effects on the one hand and unwanted tissue damage and inflammation in a variety of diseases on the other hand. Neutrophil granules comprise four principal types: primary, secondary, and tertiary granules, as well as secretory vesicles [83]. Among these, primary granules contain the highest concentrations of pro-inflammatory and antimicrobial proteins, and their release needs the strongest stimulus [83]. In contrast, the content of secretory and tertiary granules and secretory vesicles are more readily released [84]. An excessive or dysregulated release of inflammatory mediators from neutrophil granules may exacerbate the inflammatory response associated with uveitis, not only via tissue damage but also through stimulation of the adaptive immune system. The latter was shown for the short helical antimicrobial host defense peptide cathelicidin, which is released from secondary granules after neutrophil degranulation and has the ability to trigger Th17 cell differentiation and also to promote the survival of these cells in mice [85]. Th17 has been widely described as a driver of inflammatory processes in rodent models for autoimmune uveitis [86], and although Th17 cells have not directly been described in horses, matching cytokine patterns in equine iris and choroidea point to a role of Th17 cells in ERU [87]. Since the canonical pathway “neutrophil degranulation” was highly activated in ERU granulocytes after stimulation with PMA, the concept of effector cell subset differentiation and survival being triggered through neutrophil degranulation may also apply to the horse model used in our study.

One of the molecules allocated to the neutrophil degranulation pathway after PMA stimulation was CD44, a cell surface glycoprotein with elevated levels in PMA-stimulated granulocytes from ERU horses. This intracellular increase has also been observed in other models describing CD44/ERM-mediated binding of F-actin to the plasma membrane as a fundamental process for the maintenance of neutrophil morphology, migration, and nuclear degranulation [88]. Although said study described CD44 to be involved in nuclear degranulation in mice, the allocation of this molecule to the neutrophil degranulation pathway in horses might point to a similar role. CD44 was previously described to promote neutrophil adhesion as a potential ligand for Galectin-9, which is expressed in a variety of cells, including endothelial cells in blood vessels [89]. An increase of CD44 levels in PMA-stimulated granulocytes from ERU horses may therefore point to a facilitated infiltration of granulocytes into the eye, compared to CD44+ CD4+ T cells [90,91]. These T cells have been shown to be key mediators in chronic autoimmune uveitis with the ability to traverse the retina-blood barrier, releasing IL-17 and IFNγ, resulting in structural and functional damage in the process [90,91]. This was supported by the observation that disease severity in mice was reduced via administration of anti-CD44 mAb (IM7) at the early leukocyte-infiltration stage [92]. Further studies showed that CD44 levels in the iris, ciliary body, choroid, and retina are higher in patients suffering from sympathetic ophthalmia [93]. This hints towards an important role for CD44 for the transition of T cells into the diseased retina that might also apply to granulocytes.

While interpreting pathway analysis results in the hypothesis generating approach presented here, we need to keep in mind that the activation of cellular pathways is dynamic and context-dependent, and the current study represents a snapshot in early granulocyte activation in vitro. Additional kinetic studies over longer time periods could reveal the dynamic changes in pathway activity and more experiments are needed to confirm the functional relevance of the identified pathways in this study.

From the results of this study, we conclude that granulocytes from an equine model for autoimmune-mediated recurrent pan-uveitis react to different stressors in a very specific way. With a presumably pre-activated phenotype in disease, stimulation of granulocytes with IL8 predominantly promotes Class I MHC-mediated antigen processing and presentation whereas PMA promotes neutrophil degranulation. Both of these processes have previously been shown to be more readily activated in diseased state [18] and treatment with IL8 or PMA seems to preferably push the cell’s reaction towards one of these pathways. LPS on the other hand promoted processes in phospholipid biosynthesis, which was not yet described to be on standby mode in diseased state. Overall, our study underlines the potential for remarkably differentiated responses of granulocytes, offering valuable insights into their functional heterogeneity. This may support the implementation of novel concepts for diagnostics and therapy on a molecular level by targeting pathways that are activated in early stages of granulocyte activation in a T-cell-driven, sight-threatening disease.

## Figures and Tables

**Table 1:** Top enriched biological functions with predicted activation in granulocytes from horses with ERU, after stimulation with IL8, LPS, or PMA in vitro.

Category ^1^	Diseases and Biological Functions ^2^	z-Score ^3^	*p*-Value ^4^	Molecules ^5^
**IL8**				
Cell-To-Cell Signaling and Interaction	Activation of cells	1.56	3.56	AIP AMFR APOE BPI BSG CCN2 CD300LF CD37 CD81 CD84 CXCR2 ENTPD1 EPX FCER1G GBA1 GP5 HSPH1 HVCN1 IKBKB ITGB3 JAK3 LTB4R MGST1 MYD88 NPC2 NUCB2 PLAT PLSCR1 PPIF RIGI SEMA4A SLC11A1 SPTB SPTLC2 THBS1 TNFSF14 VWF
Cell-To-Cell Signaling and Interaction, Hematological System Development and Function, Immune Cell Trafficking, Inflammatory Response	Activation of leukocytes	2.17	2.86	AIP APOE BPI BSG CD300LF CD37 CD81 CD84 CXCR2 EPX FCER1G GBA1 HSPH1 HVCN1 IKBKB JAK3 LTB4R MGST1 MYD88 PLAT PLSCR1 RIGI SEMA4A SLC11A1 SPTLC2 THBS1 TNFSF14
Inflammatory Response	Inflammatory response	1.90	2.65	ALOX5AP APOE BPI BSG CD37 CD84 CXCR2 CYBA DDT EPX FCER1G HSPB1 IKBKB IRF3 ITGB3 LTB4R MSRA MYD88 NLRC4 PLAT SBDS SLC11A1 TBXAS1 THBS1 TNFSF14 TUBA4A TUBB1 TUBB2A VWF
Cell Death and Survival	Apoptosis	2.41	3.00	ACTC1 AIP ALDH3A1 AMFR APOE ARHGAP18 ASS1 BPI BSG CCDC12 CCDC47 CCN2 CIAPIN1 CKAP5 CKB COX6B1 CSTA CXCR2 CYBA DDX5 DENR EIF2B5 EIF4E ENTPD1 FBXO31 FCER1G FHL1 GBA1 GCLC GPX7 HK2 HNRNPH1 HSPB1 HSPH1 IKBKB ILKAP IRF3 ITGB3 ITM2B JAK3 KDELR1 LIMS1 MBOAT7 MRC2 MYD88 NLRC4 OAS1 PARVB PLAT PLSCR1 PPIF PPP1R2 PRDX4 PRKAR2B RABL6 RBM3 RIGI SAP18 SBDS SEMA4A SEPTIN2 SEPTIN9 SH3GLB1 SHROOM2 SNRPG SPTLC2 THBS1 TMBIM6 TNFSF14 TRAF7 TSPO TTC39C UBR2 UFL1 VPS41 VTI1A VWF
Cellular Movement	Cellular infiltration by myeloid cells	1.99	3.34	ALOX5AP AMFR APOE BSG CCN2 CD300LF CXCR2 FCER1G GBA1 HSPB1 IKBKB IRF3 ITGB3 JAK3 LTB4R MYD88 PLAT VWF
Cellular Movement, Hematological System Development and Function, Immune Cell Trafficking	Cellular infiltration by leukocytes	2.09	2.43	ALOX5AP AMFR APOE BSG CCN2 CD300LF CXCR2 FCER1G GBA1 HSPB1 IKBKB IRF3 ITGB3 JAK3 LTB4R MYD88 PLAT SPTLC2 THBS1 VWF
	Cellular infiltration by phagocytes	1.74	3.34	ALOX5AP APOE BSG CCN2 CD300LF CXCR2 FCER1G GBA1 HSPB1 IKBKB IRF3 ITGB3 JAK3 LTB4R MYD88 PLAT VWF
	Cell movement of leukocytes	1.70	2.74	ALOX5AP AMFR APOE ARHGAP18 BSG CCN2 CD300LF CD37 CD81 COMMD8 CXCR2 DDT DPYSL2 EIF4E FCER1G GBA1 HSPB1 IKBKB IRF3 ITGB3 JAK3 LTB4R MYD88 NLRC4 PLAT RIGI SBDS SEMA4A SPTLC2 THBS1 TNFSF14 VWF
	Cellular infiltration by granulocytes	1.57	3.94	ALOX5AP AMFR APOE CCN2 CD300LF CXCR2 FCER1G HSPB1 IKBKB IRF3 JAK3 LTB4R MYD88 PLAT VWF
**LPS**				
Cell morphology	Cell rounding	1.95	3.23	ITGAV ITGB8 NEDD9 PARVB PIP5K1A RAF1 SOD2
**PMA**				
Cell-To-Cell Signaling and Interaction, Inflammatory Response	Response of phagocytes	1.96	1.60	C5AR1 CD44 CLEC5A FCER1G G6PC3 ITGB3 MAP4K1 PLAUR PRKAA1 RIGI TAPBP TYROBP
	Immune response of phagocytes	1.74	1.52	CD44 CLEC5A FCER1G G6PC3 ITGB3 MAP4K1 PLAUR PRKAA1 RIGI TAPBP TYROBP
Cell-To-Cell Signaling and Interaction	Activation of cells	1.59	2.04	AMFR ANK3 APOE C1GALT1C1 C5AR1 CCN2 CD300LF CD44 CD81 CD84 CLCN7 CLEC5A CTSS DDOST DGAT1 EPX FCER1G FERMT2 FGG IKBKG ITGAV ITGB3 JAK3 KRT8 LSP1 MAGT1 MAP4K1 NAMPT NPC2 NUCB2 PLAUR PRKAA1 RIGI SLC11A1 SNAP23 SPTB TIMP2 TYROBP VWF

^1^ categories that the activated biological functions belonged to, ^2^ name of the activated biological function identified with IPA, ^3^ activation z-score (only top activated biological functions with z-score > 2 are shown), ^4^ –(log10) *p*-value for predicted activation of respective biological function, ^5^ proteins identified from stimulated granulocytes via mass spectrometric analysis, allocated to respective biological function after IPA analysis.

**Table 2:** Top enriched canonical pathways with predicted activation in granulocytes from horses with ERU, after stimulation with IL8, LPS, or PMA in vitro.

Canonical Pathways ^1^	z-Score ^2^	*p*-Value ^3^	Molecules ^4^
**IL8**			
SRP-dependent cotranslational protein targeting to membrane	2.24	1.57	RPL18 RPL19 SEC11A SEC61B SPCS2
Class I MHC-mediated antigen processing and presentation	2.14	3.07	ANAPC2 CYBA FBXO31 IKBKB MRC2 MYD88 PIK3R4 TAP2 TAPBP TRAF7 UBE2L6 UBR2 UFL1 VAMP3
TEC Kinase Signaling	2.00	1.66	ACTC1 FCER1G GNG10 ITGB3 ITGB8 JAK3 PIK3R4
**LPS**			
SRP-dependent cotranslational protein targeting to membrane	2.31	5.58	RPL13 RPL15 RPL26 RPL27A RPL35 RPL7A RPLP1 RPS13 RPS26 SEC11A SEC61A1 SPCS2
CDP-diacylglycerol Biosynthesis I	2.00	2.76	AGPAT2 CDS1 CDS2 MBOAT7
Phosphatidylglycerol Biosynthesis II (Non-plastidic)	2.00	2.63	AGPAT2 CDS1 CDS2 MBOAT7
**PMA**			
Neutrophil degranulation	3.53	7.61	ALDH3B1 C5AR1 CD44 CHIT1 CLEC5A CSNK2B CTSS DDOST DGAT1 DYNLL1 EPX FCER1G ITGAV MAGT1 MMP25 NHLRC3 NIT2 NPC2 OLR1 PLAUR QSOX1 RAB44 RAB4B RAP1A SLC11A1 SNAP23 TIMP2 TMBIM1 TYROBP
SRP-dependent cotranslational protein targeting to membrane	2.12	2.80	DDOST RPL19 RPL22 RPL9 RPLP0 RPS10 RPS9 SEC11A

^1^ name of the activated canonical pathway identified with IPA, ^2^ activation z-score (only top activated canonical pathways with z-score > 2 are shown), ^3^ –(log10) *p*-value for predicted activation of the respective canonical pathway, ^4^ proteins identified from stimulated granulocytes via mass spectrometric analysis, allocated to respective canonical pathway after IPA analysis.

## Data Availability

The mass spectrometry proteomics data have been deposited to the ProteomeXchange Consortium (http://proteomecentral.proteomexchange.org (accessed on on 15 January 2020)) via the PRIDE partner repository [94] with the dataset identifier PXD013648.

## Supplementary Materials

The following supporting information can be downloaded at: https://www.mdpi.com/article/10.3390/biomedicines12010019/s1, Table S1: Proteomics output data. Table S2: IPA comparison analysis.
